# Inflammatory Orofacial Pain Activates Peptidergic Neurons and Upregulates the Oxytocin Receptor Expression in Trigeminal Ganglion

**DOI:** 10.3390/biomedicines11092419

**Published:** 2023-08-29

**Authors:** Péter Bátor Kemenesi-Gedei, Krisztina Anna Csabafi, Gyöngyi Kis

**Affiliations:** 1 Department of Physiology, Albert Szent-Györgyi Medical School, University of Szeged, 6720 Szeged, Hungary; 2 Department of Pathophysiology, Albert Szent-Györgyi Medical School, University of Szeged, 6720 Szeged, Hungary; 3 Department of Physiology, Anatomy and Neuroscience, Faculty of Science and Informatics, University of Szeged, 6720 Szeged, Hungary

**Keywords:** orofacial pain, oxytocin, oxytocin receptor, c-Fos, CGRP, primary sensory neuron, nociception

## Abstract

The majority of orofacial pain is caused by musculoskeletal and neuropathological diseases related to inflammatory processes that lead even to transcriptional alterations in the trigeminal ganglion (TG) neurons. The hypothalamic nonapeptide oxytocin has been reported to modulate nociception via binding and activating its receptor in primary sensory neurons. The purpose of this study was to analyze the gene expression of the oxytocin receptor (OTR), c-Fos, an indicator of neuronal activity, and α-calcitonin gene-related peptide (αCGRP), a characteristic neurotransmitter of the peptidergic trigeminal primary afferents in an animal model of inflammation-induced orofacial pain. Carrageenan was unilaterally injected into the vibrissal pads of male and female adult Wistar rats. RT-qPCR was performed to analyze the levels of mRNA expression in TGs 24 h after injection. The gene expression analysis revealed higher fold changes regarding the c-Fos (mean ± S.E: ♀: 3.9 ± 0.19; ♂: 3.55 ± 0.18) and αCGRP (♀: 2.84 ± 0.13; ♂: 3.39 ± 0.47) expression levels of mRNA, and a moderate rise in the expression of the OTR mRNA (♀: 1.52 ± 0.07; ♂: 1.49 ± 0.07) was observed in comparison to both vehicle(saline)-treated and untreated controls. Our results furnish evidence for inflammation-induced activation of peptidergic neurons, and it is suggested that oxytocin modulates inflammation-induced nociception by enhancing their signaling capacity due to its elevated expression in the sensory ganglion cells, thus providing new therapies for orofacial pain relief that target the OTRs.

## 1. Introduction

Pseudounipolar nociceptors receive and process stimuli of thermal, mechanical, and chemical modalities that are damaging or potentially damaging to tissue via their specific receptors [1]. Nociceptive primary afferents can be classified into peptidergic and non-peptidergic groups based on their neurochemical markers. The peptidergic nociceptors express peptide-type neurotransmitters such as substance P (SP), calcitonin gene-related peptide (CGRP), galanin, pituitary adenylate cyclase activating polypeptide [2]. Peptidergic afferents expressing CGRP include nociceptors and high-threshold mechanoreceptors (HTMRs), both of which are involved in the innervation of the orofacial area. There may be pathophysiological conditions that alter the responsiveness of primary afferents, which manifest by increased excitability of the primary sensory neuron [3]. It has been observed that nerve endings of nociceptors become sensitized in response to locally released substances, such as prostaglandins, leukotrienes, SP, and CGRP. These sensitizing mediators set a lower threshold for nociceptor activation, but are not able to stimulate them directly. Chemosensitive nociceptors can be directly activated by endogenous substances also, such as H^+^, serotonin, histamine, and bradykinin. This stimulation leads to increased sensitivity and may be manifested in hyperalgesia or allodynia. It occurs when these chemical mediators stimulate the receptors on the nociceptive terminals, and influence the protein enzyme cascade which then upregulates the ion channels, sodium-specific nociceptive channels, and other receptors. Thus, the intracellular cascade makes the neurons more sensitive to chemical mediators and the influx of ions causes altered excitability via either de- or hyperpolarization of the neuron. In animal models of pain that are widely used in basic research, nerve injury is often induced by inflammatory adjuvants to study the sensitization and activation of primary nociceptors [4,5,6]. Eliava et al. reported that in the presence of a proinflammatory stimulus, oxytocin (OT)-expressing parvocellular paraventricular neurons (parvoPVN) are excited, and through their consequent activation, this population of OT neurons reduce the electrical activity of nociceptive Aδ- and C-type fiber-mediated wide dynamic range (WDR) neurons stimulated by the nociceptive stimuli. When bound to and stimulated by the oxytocin receptor (OTR), they can trigger G-protein-mediated signaling in the WDR neurons [7]. One possible mechanism of action is that by enhancing the activity of the phospholipase C enzyme via the OTR-coupled Gαq protein, the signaling pathway of the sensory neuron leads to an increase in the cytoplasmic Ca^2+^ ([Ca^2+^]_i_) concentration, which subsequently leads to activation of the Ca^2+^/nNOS/NO/K_ATP_ signal transduction pathway and ends in hyperpolarization of the neuron [8,9,10,11]. OT is a nonapeptide, known to be produced in the magnocellular PVN (magnoPVN) and supraoptic (SON) nuclei of the hypothalamus. Indeed, it has long been known that magnocellular oxytocinergic axons terminate in the posterior lobe of the pituitary, where OT, as a neurohormone, is stored in the Hering’s bodies of OTergic axon terminals and OTergic cells secrete OT into the capillary network of the pituitary upon appropriate stimulation. As a neurohormone, its role in smooth muscle contraction during labor and lactation has particular importance. As a neuromodulator or neurotransmitter, it also regulates emotional, mood, and behavioral processes in the brain, such as those responsible for maternal bonding [12]. Nonetheless, it is now accepted that OT plays a role in the modulation of pain transmission via its receptors. OTRs are present in the dorsal horn of the spinal cord of both the human and rat central nervous systems, as well as in the spinal trigeminal nucleus of the brainstem, in the ganglia of the peripheral nervous system (spinal, autonomic, and trigeminal), with varying densities [7,13,14]. Although it has been described before that axons from the hypothalamic level descend to superficial layers of the spinal cord [15], recent evidence suggests that the parvocellular oxytocinergic neurons project not only to the deep layers of the spinal cord but to the SON and upon activation they induce two pathways of OT action: via the circulation and via the direct synaptic influence of spinal sensory neurons. Since in the rat trigeminal ganglia (TG) lacks the blood–brain barrier [16], oxytocin can reach TG neurons via the blood circulation [17].

Relevant studies have described that orofacial inflammation leads to transcriptional alterations in trigeminal primary sensory neurons of the trigeminal nerve (cranial nerve V; these changes and the regulatory processes resulting from orofacial inflammatory pain in various TG neuron types have been investigated [18,19]. Although a huge number of novel putative genes in peptidergic neurons have been found to alter their expression suggesting that they play a crucial role in controlling orofacial inflammatory pain, a large amount of the detected genes require further verification to identify as potential targets for developing pain management strategies.

The role of OT and its receptor in orofacial pain and trigeminal sensitization have been poorly investigated. Our research aimed to investigate the effects of acute local orofacial inflammation associated with pain in the peripheral trigeminal system on the activation of neurons and OTR expression.

## 2. Materials and Methods

### 2.1. Animals

Wistar rats were maintained on a 12:12 h light/dark cycle (lights at 6:00 am) and constant temperature (21 ± 1 °C). Food and drink were available ad libitum throughout the experiment. All animals have a minimum acclimation period of 2 weeks prior to the experiments. All experiment procedures were approved by the Ethics Committee for Animal Care of the University of Szeged in accordance with the European Communities Council Directive of 24 November 1986 (86/609/EEC) under the identifier XIV/168/2022 and all efforts were made to minimize the number of animals used and their suffering. A total number of 26 male and 18 female rats were used as follows: animals were randomly assigned to carrageenan-treated (n = 6/group), saline-treated (n = 6/group), and naïve control (n = 6/group) groups by sex, and n = 4 of carrageenan-treated male animals with one and n = 4 of carrageenan-treated male animals with three days of survival for morphological experiments.

### 2.2. Establishment of the Animal Model

An appropriate animal model of acute local inflammatory pain was created [5,18] using 9–11 week old (200–300 g) adult female (nulliparous, non-pregnant, and non-lactating) and male Wistar rats. To induce inflammation the animals received unilateral subcutaneous (s.c.) injections of a mixture of κ- and λ-carrageenans (100 µL, of 2% *w*/*v*; Sigma, St. Louis, MO, USA, C1867) or 100 µL 0.9% NaCl (saline) into their vibrissal pads under pentobarbital (45 mg/bodyweight kg, i.p.) [19] anesthesia.

### 2.3. Orofacial Operant Test

The orofacial operant test was performed according to Cha et al., with slight modifications [20]. The animals (n = 6/groups by sex) initially underwent one week of pre-treatment adaptation training utilizing the Ugo Basile^®^ Orofacial Stimulation (UBOS) Test System (Comerio, VA, Italy). The animals were placed into the apparatus’s special cage for 30 min and the stimulation tests were performed. The experiments were performed every day at the same time, in the same environment, and for the same duration. In the apparatus cage, the animals had access to a drinker containing a reward (15% sucrose solution) through a drinking window. They voluntarily placed their head into the window to enter and acquire the reward. During habituation, animals were trained to access the reward without stimulation. Upon testing the sensitivity, a panel with metal filaments (stimulation module) was placed in the drinking window and as the rats entered to acquire the sweetened water, their whisker pads contacted the wires (mechanical stimulus). In the Ugo Basile^®^ apparatus, the time the head spent in the drinking window (break duration) and the counts of drinking attempts (break count) were automatically and continuously detected and recorded with a built-in infrared photo-beam module. After recording the control values using the stimulation module, animals were subjected to carrageenan treatment and 24 h later the mechanical sensitivity was tested again. The measured data were evaluated using ORO-Software1.3^®^, and the total break duration per break count (mean break duration) was calculated for each animal from the recorded values before (control) and after the treatment.

### 2.4. Morphological Experiments

Animals used for immunohistochemistry were anesthetized with pentobarbital (45 mg/bodyweight kg, i.p.) [19] 24 h after carrageenan treatment (n = 4) or three days after receiving s.c. 100 µL retrograde tracer True Blue (TB) (Sigma, T5891) in distilled water (2–3%) into their whisker pads (n = 4). The animals under anesthesia were then transcardially perfused with 0.9% NaCl followed by fixative (4% formaldehyde in 0.1 M phosphate buffer saline (PBS); pH 7.4) prior to removal of the TGs. TGs were then dissected from the skull base and stored in vials containing the same fixative for an additional 3 h, and cryoprotected for 2 days in 0.1 M phosphate buffer containing 30% glucose. Slices of the fixed TGs with a thickness of 17 µm were placed on slides with a cryostat. For TB visualization, the frozen sections were directly analyzed without any further histochemical processes. For immunohistochemistry, PBS containing 0.3% Triton X-100 was used as a solvent for all the immunohistochemical processes. After incubation with 1% of normal donkey serum for 20 min, the TG sections were incubated with primary antibodies anti-OTR (1:400, Alomone, Jerusalem, Israel, AVR-013) and anti-CGRP antibody (Sigma, C7113) for 24 h at R.T., and then with secondary antibodies Cy3-conjugated donkey anti-rabbit (1:500, Jacksons ImmunoResearch, West Grove, PA, USA, code no. 711-167-003) and anti-mouse Alexa Fluor^®^ 488-conjugated antibody (1:500, Jacksons ImmunoResearch, code no. 711-545-020) for 2 h at R.T. Washes between each incubation period and at the end were performed in PBS each time. Sections were covered with ProLong™ Gold Antifade Mountant with DNA Stain DAPI (Invitrogen™, Waltham, MA, USA, Thermofisher Scientific, Waltham, MA, USA, cat. no. P36935). Images were captured with Zeiss LSM 700 confocal laser scanning microscope.

### 2.5. RNA Extraction and Quantitative Real-Time Polymerase Chain Reaction (qRT-PCR)

Twenty-four hours after treatment, animals were tested by UBOS as described above, and at the end of the test, qRT-PCR was used to measure the target mRNAs in the TGs ipsilateral to the injection. The saline-treated animals (n = 6/groups by sex) and those used for naïve control samples (n = 6/groups by sex) were not subjected to the UBOS test. Rats were deeply anesthetized with pentobarbital (45 mg/bodyweight kg, i.p.) [19], and after exsanguation, TGs were removed. RNA extraction was performed according to the manufacturer’s instructions using TriXtract^TM^ reagent (G-Biosciences, St. Louis, MO, USA, cat. no. 786-652). Briefly, after the homogenization of tissue samples in TriXtract^TM^ reagent, the RNA content was separated into an aqueous phase with the addition of chloroform. The precipitation with isopropyl alcohol was followed by a washing with 70% ethanol and then the RNA pellet was dissolved in RNase-free water. The quantity and quality were verified by using a Genova Nano micro-volume spectrophotometer (Jenway, London, UK) at an optical density of 260 and 260/280 nm, respectively; all samples that were used for further analysis exhibited an absorbance ratio in the range of 1.6–2.0. Equal amounts of the RNA were used to synthesize cDNA in each experiment using an iScript cDNA synthesis kit (Bio-Rad, Hercules, CA, USA, cat. no. 1708891). The control TGs from naïve animals were processed together as a single sample, whereas the ipsilateral TGs from the saline-treated and carrageenan-treated animals were processed separately. PCR was carried out in a thermocycler (Bio-Rad CFX96TM Optics Module) preparing triplicates of reactions of 10 µL in volumes using iQ™ SYBR^®^ Green Supermix (Bio-Rad, cat. no. 1708882). The thermal cycling condition included an initial denaturation step at 95 °C for 30 s and 40 cycles of denaturation at 95 °C for 10 s, annealing at 57 °C for 30 s, and extension at 72 °C for 20 s. Finally, the amplicons were subjected to melting curve analysis. A pair of primers previously designed by Bangaru et al., was applied in-house to amplify a 142-bp fragment of the glyceraldehyde 3-phosphate dehydrogenase (GAPDH) mRNA [21]. Expression of GAPDH as a housekeeping gene (loading control) was determined from the same set of samples to use as an internal normalizer. Further primer pairs were designed using the National Centre of Biotechnology Information (NCBI) reference sequence database (https://www.ncbi.nlm.nih.gov/Entrez (accessed on 16 November 2021)) to amplify a 140-bp fragment of the OTR, a 97-bp fragment of c-fos and a 90-bp fragment of α-CGRP (also known as calcitonin related polypeptide alpha, CALCA) mRNA (Table 1). A no-template was used as a negative control where RNAse-free water was added instead of cDNA. The threshold cycle values (Ct) were used as reference points for calculating relative gene expression. The comparative Ct method, also known as Δ∆Ct method [22] was implemented to achieve relative quantification. 2^−Δ∆Ct^ values were used to calculate fold changes in target gene expression using control groups as normalizers.

### 2.6. Statistical Analysis

To analyze the results and to determine the differences between the control values and the values after treatment regarding the operant test, Shapiro–Wilk test for normality and pairwise comparison, (Student *t*-test) were used.

Statistical comparisons were conducted using one-way analysis of variance (ANOVA), followed by Tukey’s post hoc test to compare mRNA expressions of the naïve controls and the saline- or carrageenan-treated animals using SigmaPlot 12.0 software. Operant assay results are presented as mean ± S.D. and mRNA expression data (2^−Δ∆Ct^) from quantitative PCR analysis are presented as mean ± S.E.

## 3. Results

### 3.1. Morphological Experiments

#### 3.1.1. True Blue Tracer Injection

**Figure 1:**
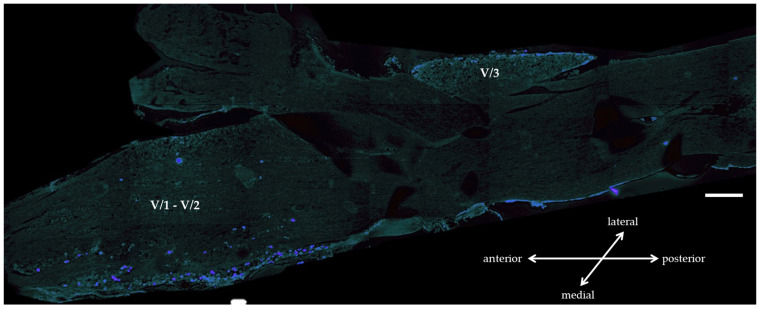
Representative photomicrograph of a trigeminal ganglion horizontal section from the TB-tracer injected group. Positive TB-labelled (blue) neurons are present in TG V/1 and V/2 regions. V/1: ophthalmic region, V/2: maxillary region, V/3: mandibular region. Scale bar = 500 µm.

TB tracer was injected into the whisker pad of the animals to map the cell bodies of the subcutaneous primary sensory afferents of the whisker pad in the trigeminal ganglion with this retrograde labeling. Three days after the injection the cells showing blue labeling (positive) were localized in predominant amounts to regions V/1 (projection of the ophthalmic nerve) and V/2 (projection of the maxillary nerve) of the TG (Figure 1).

#### 3.1.2. Immunohistochemistry of OTR and CGRP Expression in TG

**Figure 2:**
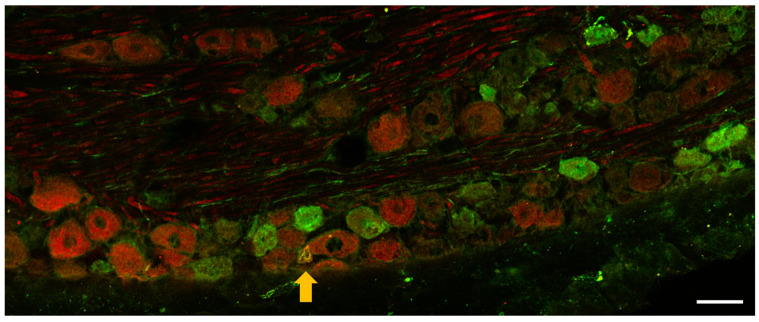
Representative fluorescence photomicrographs illustrate OTR-positive (red) and CGRP-positive (green) neurons in TG 24 after carrageenan injection into the whisker pads. The orange arrow points to co-localization. Scale bar 50 µm.

Immunohistochemistry aimed to observe OTR cells in TG. We wanted to investigate whether OTR-positive cells overlap with the peptidergic nociceptors that are typically activated in inflammatory pain processes. To detect the peptidergic cells, we targeted their characteristic neurochemical marker CGRP. OTR-positive cells, CGRP-positive cells, and cells showing OTR-CGRP colocalization were poorly detectable 24 h after carrageenan treatment in the TG (Figure 2). OTR positivity in naïve control samples could not be detected with sufficient reliability, likely because the expression level was very close to the detection limit.

### 3.2. Orofacial Operant Test

**Figure 3:**
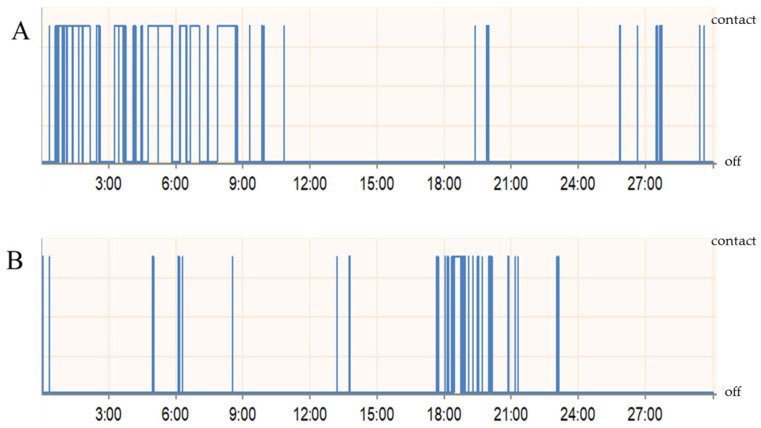
Two sample traces show the automatic recordings of drinking behavior in a 30-min testing session during control registration (**A**) and 24 h after carrageenan treatment (**B**) in a rat. Mechanical stimulation with filaments was implemented in both cases.

**Figure 4:**
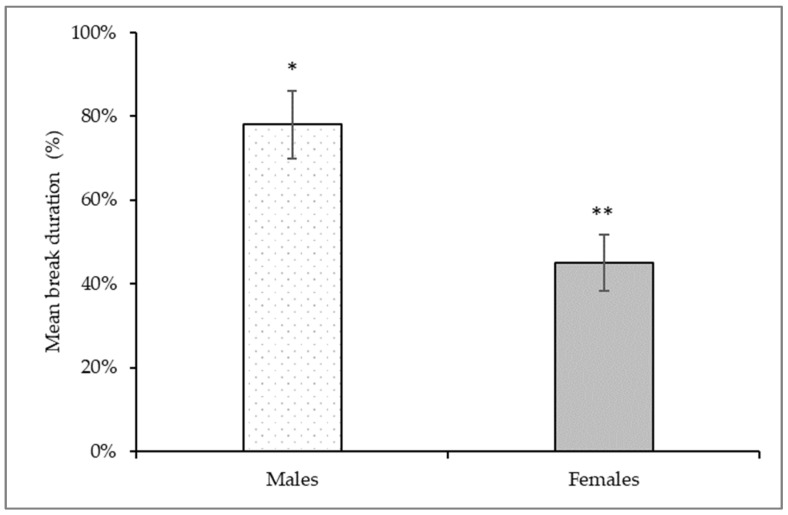
Changes in the mean break duration during orofacial operant test. Mechanical stimulus was applied bilaterally to the female and male vibrissal pads before and after carrageenan treatment. The mean break duration decreased by 22% in males and 55% in females with orofacial inflammation. Data are expressed as means ± S.D. (n = 6/groups). * Significant difference *p* < 0.05 compared to control values, ** Significant difference *p* < 0.01 compared to control values.

To assess the nociceptive response following carrageenan-induced inflammation, the sensitivity of the rat’s orofacial region to mechanical stimuli was examined using the UBOS test. In general, for most animals, there was a significant change in the time spent in the drinking window and the number of drinking sessions after the treatment (Figure 3). The values of total break duration per break duration count, i.e., the mean break duration values were analyzed. Changes were compared to the control registrations before carrageenan treatment and the results were averaged and presented as percentages. The mean time spent in the drinking window was significantly reduced by an average of 22% for males and 55% for females (Figure 4).

### 3.3. Transcriptional Analyses

We investigated the expression patterns 24 h after the saline administration and in the case of the carrageenan-treated animals even following the UBOS tests, as described in the Section 2.

#### 3.3.1. Analysis of the c-Fos mRNA Expression

**Figure 5:**
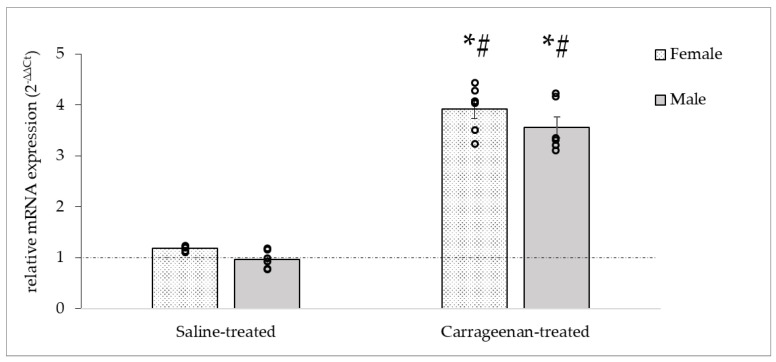
Relative expression of c-Fos mRNA in female and male TG. Carrageenan administration caused a significant elevation in the expression of c-Fos mRNA ipsilateral to the injection both in female and male groups compared to naïve controls (relative mRNA expression = 1, dashed line). Data are expressed as means ± S.E., circles represent individual data (n = 6/groups). * Significant difference (*p* < 0.05) compared to naïve controls, # Significant difference (*p* < 0.05) compared to saline-treated groups.

The relative mRNA expression changes in c-Fos were measured to verify the neuronal activation. The saline-treated groups showed no increase compared to the naïve control group in females, but in the carrageenan-treated groups a significant increase in relative c-Fos mRNA expression was observed in both female and male groups in the ipsilateral TGs compared to naïve controls (♀: 3.9 ± 0.19; ♂: 3.55 ± 0.18) (Figure 5). The statistical analysis indicated significant differences between groups by treatment (DF = 3, F = 52.94, *p* < 0.05), but not by sexes.

#### 3.3.2. Analysis of the CALCA mRNA Expression

**Figure 6:**
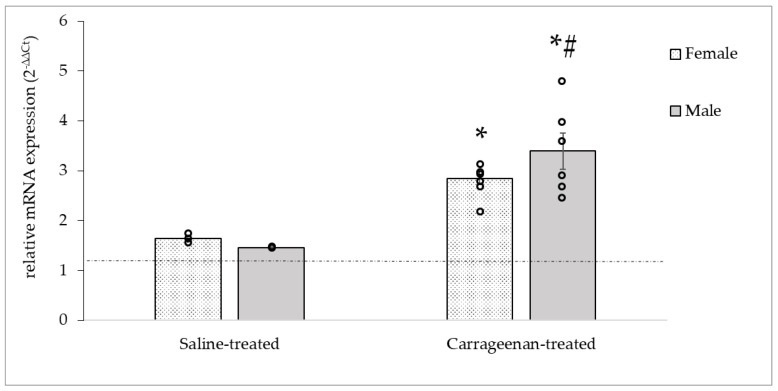
Relative expression of CALCA mRNA in female and male TG. Determination of CALCA mRNA levels showed elevation in both sexes and treatments compared to naïve control (relative mRNA expression = 1, dashed line). Carrageenan injection provoked a significant increase in gene expression TGs compared to naïve controls, and in males compared to saline-treated groups 24 h after the treatment. Data are presented as mean ± S.E., circles represent individual data (n = 6/groups). * Significant difference (*p* < 0.05) compared to naïve controls, # Significant difference (*p* < 0.05) compared to saline-treated groups.

The inflammation-induced alterations of the CALCA mRNA expression are presented in Figure 6. In both female and male saline-treated groups the CALCA expression was elevated compared to the naïve control groups (♀: 1.6 ± 0.05; ♂: 1.48 ± 0.01). However, as expected, carrageenan injection resulted in a significant increase in the CALCA gene expression in TG cells than that obtained from saline-treated neurons 24 h after treatment in both sexes (♀: 2.84 ± 0.13; ♂: 3.39 ± 0.47). There was a statistically significant difference between groups (DF = 3, F = 32.29, *p* < 0.05) without sex difference.

#### 3.3.3. Analysis of the OTR mRNA Expression

**Figure 7:**
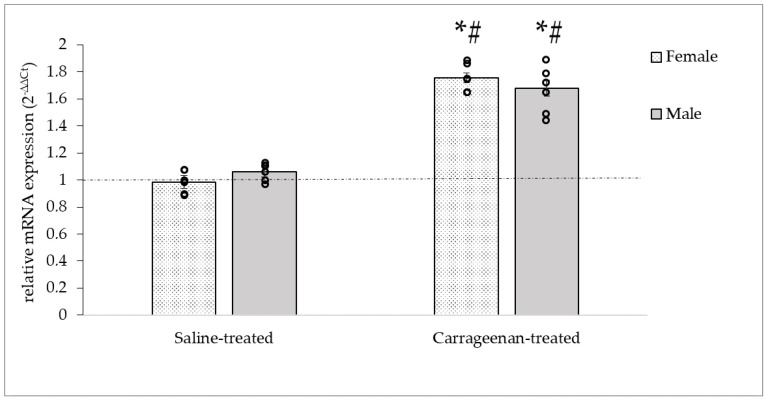
Relative expression of OTR mRNA in female and male TG. Note the increase in mRNA expression only in the carrageenan-treated groups of both sexes relative to naïve controls (relative mRNA expression = 1, dashed line). Data are presented as mean ± S.E., circles represent individual data (n = 6/groups). * Significant difference (*p* < 0.05) compared to naïve controls, # Significant difference (*p* < 0.05) compared to saline-treated groups.

The level of OTR mRNA in TGs showed a significant increase after the carrageenan treatment compared to carrageenan-treated groups (♀: 1.52 ± 0.07; ♂: 1.49 ± 0.07) in both sexes according to the statistical analyses (DF = 3, F = 44.54, *p* < 0.05), but no difference between sexes. No change in OTR mRNA expression was observed in either sex after administration of saline (Figure 7).

## 4. Discussion

One of the animal models commonly used to study trigeminal inflammatory pain is the carrageenan-induced inflammatory orofacial pain model [5]. The adjuvant is injected into the whisker pads subcutaneously which induces activation of trigeminal nerve afferents by locally induced inflammation. Injection of the retrograde tracer True Blue into the same area, which passed along the trigeminal nerve branches from the periphery to perikarya and stained the cytoplasms with fluorescent blue resulted in labeling of the cells to reveal the concerned regions in trigeminal ganglion. Our results confirmed the involvement of the trigeminal nerve V/2 branch, i.e., the maxillary nerve, which projects to the corresponding V/2 region in the ganglion. However, we could observe fluorescent staining in the V/1 region, which may be explained by the gaps between the satellite cells that surround the neurons in the TG and the fenestrated endothelium of the vasculature, which allows the staining to reach nearby cells in the ganglion [2,23]. Moreover, we cannot exclude that the labeling agent is transferred to adjacent axons running in parallel during transport; the dye injected into the meningeal branch of the mandibular division, i.e., the spinosus nerve, retrogradely spreading also showed a heterogeneous distribution in the V/2 TG regions in addition to V/3 (mandibular branch) where it was expected [24]. Our immunohistochemical analysis identified OTR- and CGRP-positive cells in the TG. The neurochemical characterization of TG or dorsal root ganglion cells expressing OTR has been described previously [25]. In addition to our results showing OTR-CGRP colocalization, OTR staining has been reported in larger-sized Aδ peptidergic afferents, and in about 1/3 of small-sized C-type primary afferents [17]. Nonetheless, it has been revealed that about 80% of cells expressing OTR are CGRP positive [14], while about 10% of cells expressing CGRP express OTR [26].

Carrageenan is known to be able to sensitize primary afferents in addition to activating them [18]. This increased sensitivity to different stimuli is usually associated with easily detectable behavioral changes in the animal. Consistent with other experimental observations, we could notice behavioral alterations following carrageenan treatment. One characteristic movement was, besides rubbing the face, that during the operant test, the animals tilted their heads to access the drinker in the window trying not to contact the orofacial area with the metal filaments. In the hypersensitivity test, our numerical data confirmed that the animals’ tolerance to the stimulus was significantly reduced. The nocifensive behavior of females was more significant than that of males. The difference between the sexes may be explained by different transcriptional levels of responses to pain. Indeed, genes such as ion channels affecting synaptic transmission, modulators of the inflammatory process, and modulators of channels involved in mechanical sensitivity are up- or down-regulated differently in response to pain [27].

Carrageenan, a member of the polysaccharide family, can induce an acute, transient inflammatory milieu in the affected area by stimulating the Toll-like receptor 4-coupled signal transduction pathways. It has been suggested that carrageenan-induced inflammation, and thus stimulation of primary afferents, manifest most prominently within the first day after administration [5]. In our experimental process mRNA expression assays examined how the expression of the neuronal activity marker, transcription factor c-Fos [28] changed following the inflammation. We observed an increase in gene expression that reflects neuronal activity following the adjuvant injection. Although, this almost four-fold change was below the increase in c-Fos mRNA expression levels that has previously been observed by others [29,30,31]. Demartini et al. induced formalin-induced orofacial inflammation in the rat by measuring this much higher relative expression in ipsilateral TGs removed within hours of treatment [29]. The more modest increase in c-Fos expression we observed can be explained by the time lag between inflammatory induction and mRNA extraction. c-Fos peaks in the first few hours after the painful stimulus and then gradually returns to lower levels [32]. Carrageenan induces an acute, transient, whereas other irritants (e.g., formalin and complete Freund’s adjuvant (CFA)) induce a more prolonged inflammation. In addition to the length of the time window mentioned above, the nature of the adjuvant used may also influence the expression of certain target genes.

CGRP, a vasoactive substance specific to peptidergic nociceptors, plays a crucial role in the hypersensitivity that accompanies inflammatory processes. CGRP occurs in two isoforms in the rat nervous system. β-CGRP is predominant in the enteric nervous system and motor neurons, whereas α-CGRP (also known as CALCA) expression dominates in the central nervous system and somatosensory fibers [2,33]. In our experiments, we measured significant CGRP expression in the inflammation-induced ipsilateral TG, providing evidence for a peptidergic neuronal response to inflammation. Interestingly, we also measured elevated CGRP mRNA levels in physiological saline-treated animals, albeit to a lesser extent. CGRPergic nociceptors may differ not only in morphology but also in the type of stimuli that activate the receptors. Small pain-sensing cells are unmyelinated, C-type nociceptor afferents, and the medium-sized cells with thin myelin sheaths are Aδ-type nociceptor afferents with free nerve endings in the dermis. The C- and Aδ-types of low-threshold mechanoreceptors (LTMRs) do not express CGRP [34,35]. Among the high-threshold mechanoreceptors (HTMRs), the medium-size peptidergic Aδ HTMRs with their special morphology and “lasso-like” circumferential end branches innervate the hair follicles one by one. Despite their different morphology, activation of these CGRPergic cell types results in an escape response of the animal and a change in behavior to avoid the stimulus, which may support their nocifensive function [2,36]. Thus, CGRPergic nociceptors can be activated by different stimuli. Pinching or needle pricking of the skin is a putative stimulus for Aδ-type HTMRs supplying the affected area, pulling a hair is a putative stimulus for Aδ fibres with free nerve endings, while extreme temperature changes or capsaicin as a chemical for C-type CGRPergic afferents [2,27]. Nonetheless, we speculate that members of the Aδ-HTMR CGRPergic subpopulation may respond to mechanical stimuli such as the short one provoked by saline injection to promote their CGRP expression, as well as the mechanical stimulus of UBOS test in the carrageenan-treated animals to elevate the “early-gene marker” c-fos expression. Thus, we suggest that these processes further enhanced the transcriptional response of proinflammatory carrageenan in both sexes.

In our experiments, we demonstrated that carrageenan-induced orofacial pain increased OTR mRNA expression in the TG. In animal models of inflammatory pain induced with a more potent proinflammatory adjuvant, CFA, a rapid up-regulation of OTR was associated with a more than 10-fold increase in OTR protein expression compared to baseline OTR levels prior to proinflammatory treatment [7,14]. Nevertheless, treatment with Non-Steroid Anti-Inflammatory Drugs (NSAIDs) attenuated the analgesic effect of OT through the reduction in interleukins that promote OTR up-regulation [13]. In addition, it has been described that OT released from hypothalamic descending parvoPVN fibers can mediate a rapid but not sustained analgesic effect via direct chemical synapses. In contrast to the central pathway, activation of the nociceptor ganglion cells by the circulating OT through the OTR may result in a slower but more prolonged analgesic effect [7].

The most common type of chronic pain associated with inflammation and affecting the trigeminal system is migraine, in the pathophysiology of which a key role is attributed to the increased excitability of CGRPergic neurons in the trigeminal-vascular system [17,33]. Approximately 50% of human TG neurons show CGRP positivity [37]. The role of CGRP in modulating nociception and in the transmission of nociceptive stimuli from the periphery to the central nervous system is of great importance. In the prevention of migraine attacks or, if exacerbation has occurred, in the termination of the attack, antimigraine drugs targeting CGRP or the CGRP release from nerves may be preferred. Nonetheless, there have already been human implications of OT in the treatment of migraine headache. A publication in 2017 by Tzabasis et al. reported that intranasal OT was able to reduce the frequency and intensity of migraine attacks in patients with both chronic migraine and high-frequency episodic migraine. However, per os administration of exogenous OT for analgesic purposes is ineffective, as the small nonapeptide is rapidly degraded in the gastrointestinal system [13].

In summary, we conclude that the carrageenan-induced orofacial pain was accompanied by the activation of peptidergic neurons, but it remains to reveal what proportion of each subpopulation is involved in the process, presumably due to the mechanical interventions in our experiments in particular the Aδ-HTMR CGRPergic neurons had a noteworthy role. The elevated OTR expression in sensory neurons may contribute to inflammatory processes and nociceptive sensitization underlying orofacial pain via the modulation of neuronal excitability. These findings open up intriguing new avenues for investigating the function and mechanisms of action of OT in neuroinflammation in different models of trigeminal sensitization, as well as its potential clinical utility.

## Figures and Tables

**Table 1:** Primer sequences used for mRNA expression studies.

Target Gene	Primer Pairs (5′→3′)	Reference Sequence
GAPDH	^1^ Fw: AGACAGCCGCATCTTCTTGT ^1^ Rev: TGATGGCAACAATGTCCACT	NM_017008.4
CALCA	Fw: GCTGCCCAGATCAAGAGTCA Rev: ACCTGGTGAGCGATGACTTG	NM_017338.2
c-Fos	Fw: GGGAGGACCTTATCTGTGCG Rev: TCTCCGGAAGAGGTGAGGAC	NM_022197
OTR	Fw: TTCTTCTGCTGCTCTGCTCGT Rev: TCATGCTGAGATGGCTGAGA	NM_012871.3

^1^ Obtained from Bangaru et al. [21].

## Data Availability

The data that support the findings of this study are available from the corresponding author G.K., upon reasonable request.
